# Relationship between ETS Transcription Factor ETV1 and TGF-β-regulated SMAD Proteins in Prostate Cancer

**DOI:** 10.1038/s41598-019-44685-3

**Published:** 2019-06-03

**Authors:** Sangphil Oh, Sook Shin, Hoogeun Song, Joseph P. Grande, Ralf Janknecht

**Affiliations:** 1 University of Oklahoma Health Sciences Center, Department of Cell Biology, Oklahoma City, OK 73104 USA; 2 Stephenson Cancer Center, Oklahoma City, OK 73104 USA; 3 Mayo Clinic, Department of Laboratory Medicine and Pathology, Rochester, MN 55905 USA; 4 University of Oklahoma Health Sciences Center, Department of Pathology, Oklahoma City, OK 73104 USA

**Keywords:** Prostate cancer, Mechanisms of disease

## Abstract

The ETS transcription factor ETV1 is frequently overexpressed in aggressive prostate cancer, which is one underlying cause of this disease. Accordingly, transgenic mice that prostate-specifically overexpress ETV1 develop prostatic intraepithelial neoplasia. However, progression to the adenocarcinoma stage is stifled in these mice, suggesting that inhibitory pathways possibly preclude ETV1 from exerting its full oncogenic potential. Here we provide evidence that TGF-β/SMAD signaling represents such an inhibitory pathway. First, we discovered that ETV1 forms complexes with SMAD4. Second, SMAD2, SMAD3 and SMAD4 overexpression impaired ETV1’s ability to stimulate gene transcription. Third, TGF-β1 inhibited ETV1-induced invasion by benign RWPE-1 prostate cells. Fourth, increased expression of SMAD3 and SMAD4 was observable in prostates of *ETV1* transgenic mice. Conversely, we found that ETV1 may enhance TGF-β signaling in PC3 prostate cancer cells, revealing a different facet of the ETV1/TGF-β interplay. Altogether, these data provide more insights into the regulation and action of ETV1 and additionally suggest that TGF-β/SMAD signaling exerts its tumor suppressive activity, at least in part, by curtailing the oncogenic potential of ETV1 in prostatic lesions.

## Introduction

The oncogenic transcription factor ETS variant 1 (ETV1) becomes overexpressed in many prostate tumors by chromosomal translocations involving the *ETV1* gene and androgen-responsive promoters^1–4^ or by loss of its negative regulator, the ubiquitin ligase COP1^5,6^. Overexpression of ETV1 increased migration and invasion by benign prostate cells^2,3,7–9^, stimulated androgen metabolism^10^, and correlated with higher disease recurrence and reduced survival in prostate cancer patients^10,11^. In addition, a small molecule inhibitor of ETV1 suppressed prostate cancer cell proliferation *in vitro* and *in vivo*^12,13^. All this suggests that ETV1 is required for efficient growth and aggressiveness/metastasis during prostate cancer progression.

In line with this, ETV1 overexpression can lead to increased transcription of the matrix metalloproteinase 1 (*MMP1*) and *MMP7* genes^14–16^, which both are important for cancer cell invasion. Also, transgenic mice overexpressing ETV1 in the prostate presented with prostatic intraepithelial neoplasia (PIN), yet deficiency of the tumor suppressor PTEN, which is commonly observed in human prostate tumors, was additionally required for the development of prostate adenocarcinomas in *ETV1* transgenic mice^2,10,11,17^. Interestingly, transcriptional activity of ETV1 can be vastly enhanced by mitogen-activated protein kinase signaling pathways through posttranslational modification of ETV1^14,18–21^, suggesting that not only overexpression, but also stimulation of its transcriptional potential may be involved in facilitating ETV1’s oncogenic role.

One unresolved question is why *ETV1* transgenic mice did not progress from PIN to the adenocarcinoma stage. A similar question related to the homozygous deletion of the tumor suppressor PTEN in the prostate of mice, which led to PIN, but only after a long latency induced adenocarcinomas that rarely metastasized^22,23^. However, deletion of SMAD4 in addition to PTEN invariably resulted into the development of metastatic, lethal prostate cancer at an early age, whereas SMAD4 ablation on its own reportedly did not cause any prostatic lesions^24^. These data suggested that SMAD4 is a barrier that can prevent progression of prostate tumorigenesis. Notably, SMAD4 is a downstream effector of transforming growth factor β (TGF-β), a cytokine with tumor suppressive activity^25^. TGF-β induces the phosphorylation of SMAD2 and SMAD3 at the plasma membrane, which causes their association with SMAD4 and translocation to the cell nucleus where these DNA-binding proteins regulate the activity of a variety of genes^26,27^. Here, we explored if TGF-β and SMAD proteins might also repress the oncogenic potential of ETV1.

## Results

### Interaction of ETV1 with SMAD proteins

**Figure 1:**
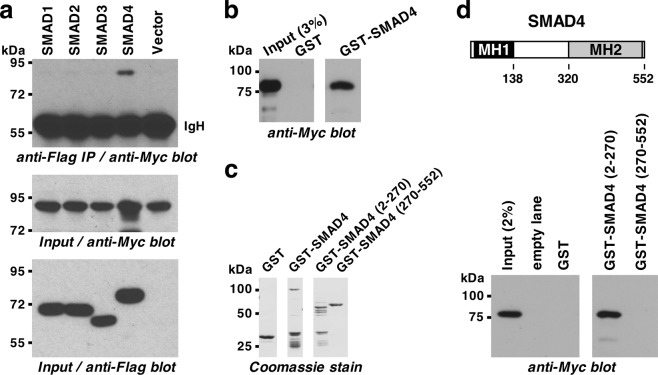
Binding of SMAD4 to ETV1. (**a**) 6Myc-tagged ETV1 was coexpressed with indicated Flag-tagged SMAD proteins in 293T cells. Immunoprecipitation (IP) was done with anti-Flag antibodies followed by anti-Myc western blotting (top panel). The bottom two panels show input levels of 6Myc-ETV1 and Flag-tagged SMAD proteins, respectively. IgH, immunoglobulin heavy chain. Blot on top was derived from a different gel than the other two blots. (**b**) Pull-down assays with GST or GST-SMAD4. Bound 6Myc-ETV1 was revealed by anti-Myc western blotting. Shown are two different parts of the same blot. (**c**) Coomassie-stained polyacrylamide gel of utilized GST-fusion proteins revealing comparable protein amounts. Shown are three different parts of the same gel. (**d**) Binding of 6Myc-ETV1 to SMAD4 truncations was assessed in GST pull-down experiments (bottom); shown are two different parts of the same blot. Sketch of SMAD4 is shown on top. Uncropped images for all four panels are presented in Supplementary Fig. S1.

To study a potential interaction of ETV1 with SMAD proteins, we coexpressed Myc-tagged ETV1 with Flag-tagged SMAD proteins and performed immunoprecipitations with anti-Flag antibodies. Any coprecipitated ETV1 was then detected by anti-Myc western blotting (Fig. 1a and Supplementary Fig. S1). Indeed, ETV1 coimmunoprecipitated with SMAD4, but not with SMAD1, SMAD2 or SMAD3. We then confirmed that bacterially expressed and purified GST-SMAD4, but not a comparable amount of the GST moiety, also interacted with ETV1 (Fig. 1b,c), suggesting that SMAD4 and ETV1 directly bind to each other. Moreover, we determined that the N-terminal half of SMAD4, which encompasses its DNA-binding MH1 domain^26^, was responsible for binding ETV1 (Fig. 1c,d).

**Figure 2:**
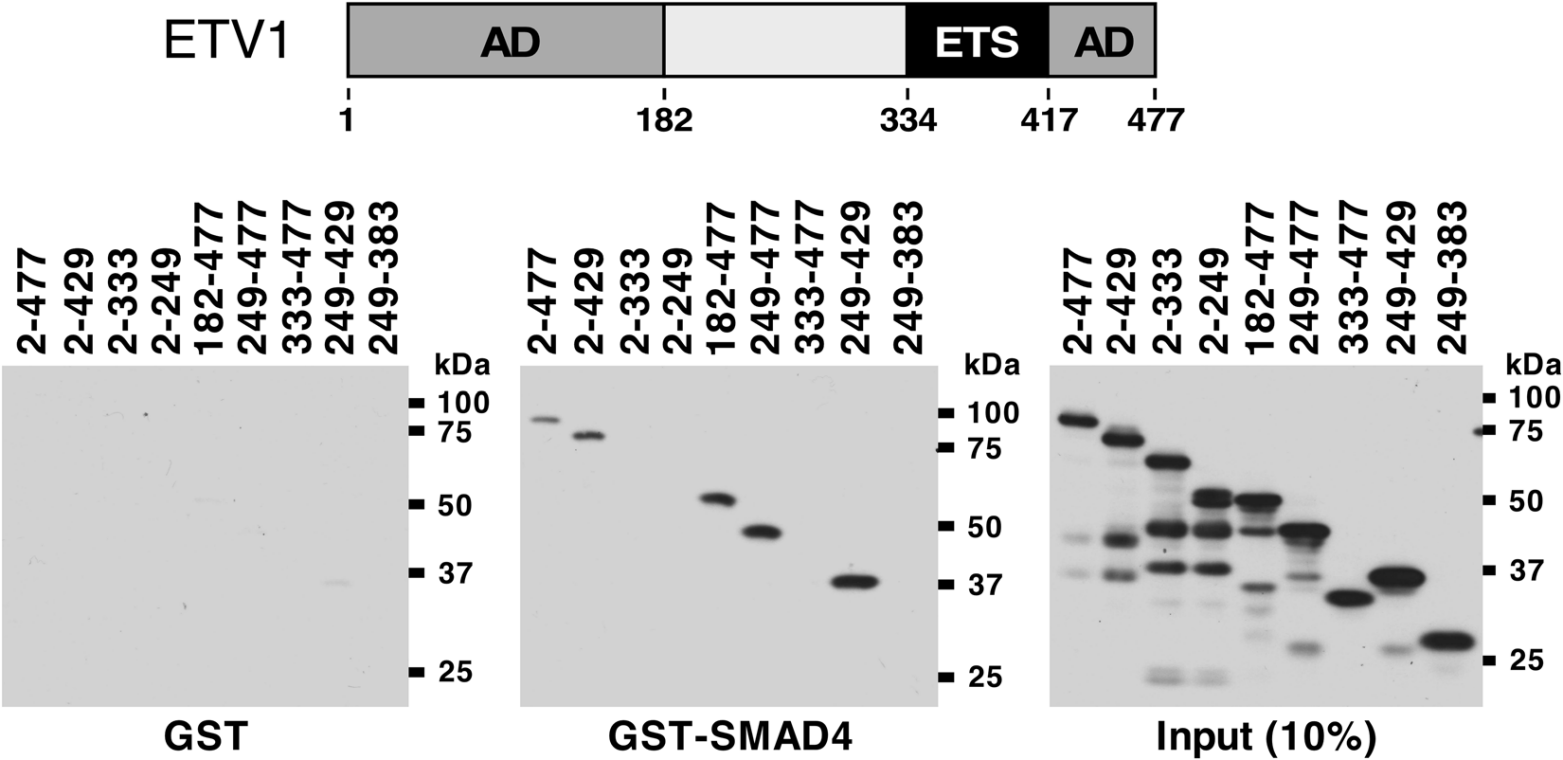
Identification of the SMAD4 interaction domain in ETV1. The top shows a scheme of ETV1 outlining its two activation domains (AD) and the DNA-binding ETS domain. Indicated 6Myc-tagged ETV1 amino acids were expressed in 293T cells and utilized for *in vitro* binding assays with GST (bottom left) or GST-SMAD4 (bottom middle). Bound ETV1 truncations were revealed by anti-Myc western blotting. The bottom right panel shows input levels for the various ETV1 truncations. The shown blots were derived from three different gels that were simultaneously processed in the same manner for western blotting and exposed to film at and for the same time. Full-length blots are presented in Supplementary Fig. S1.

Conversely, we examined which amino acids in ETV1 are required for its interaction with SMAD4. Truncating ETV1 from the C-terminus down to amino acid 429 (ETV1 2–429) did not impact on the binding to SMAD4, but further truncation down to amino acids 333 or 249 abolished this interaction (Fig. 2 and Supplementary Fig. S1). Likewise, we truncated ETV1 from the N-terminus and found that the first 248 amino acids were dispensable for SMAD4 binding (see 182–477 and 249–477 in Fig. 2). However, the 333–477 truncation did not bind to SMAD4. This suggested that amino acids 249–429 mediate the interaction with SMAD4. And indeed, ETV1 amino acids 249–429 were sufficient for binding to SMAD4, whereas amino acids 249–383 were not (Fig. 2). Overall, these data indicate that neither the N- nor C-terminal ETV1 activation domain is required for binding to SMAD4, whereas both the DNA-binding ETS domain and amino acids 249–333 of ETV1 are needed for establishing an ETV1-SMAD4 complex.

### Inhibition of ETV1 transcriptional activity by SMAD proteins

**Figure 3:**
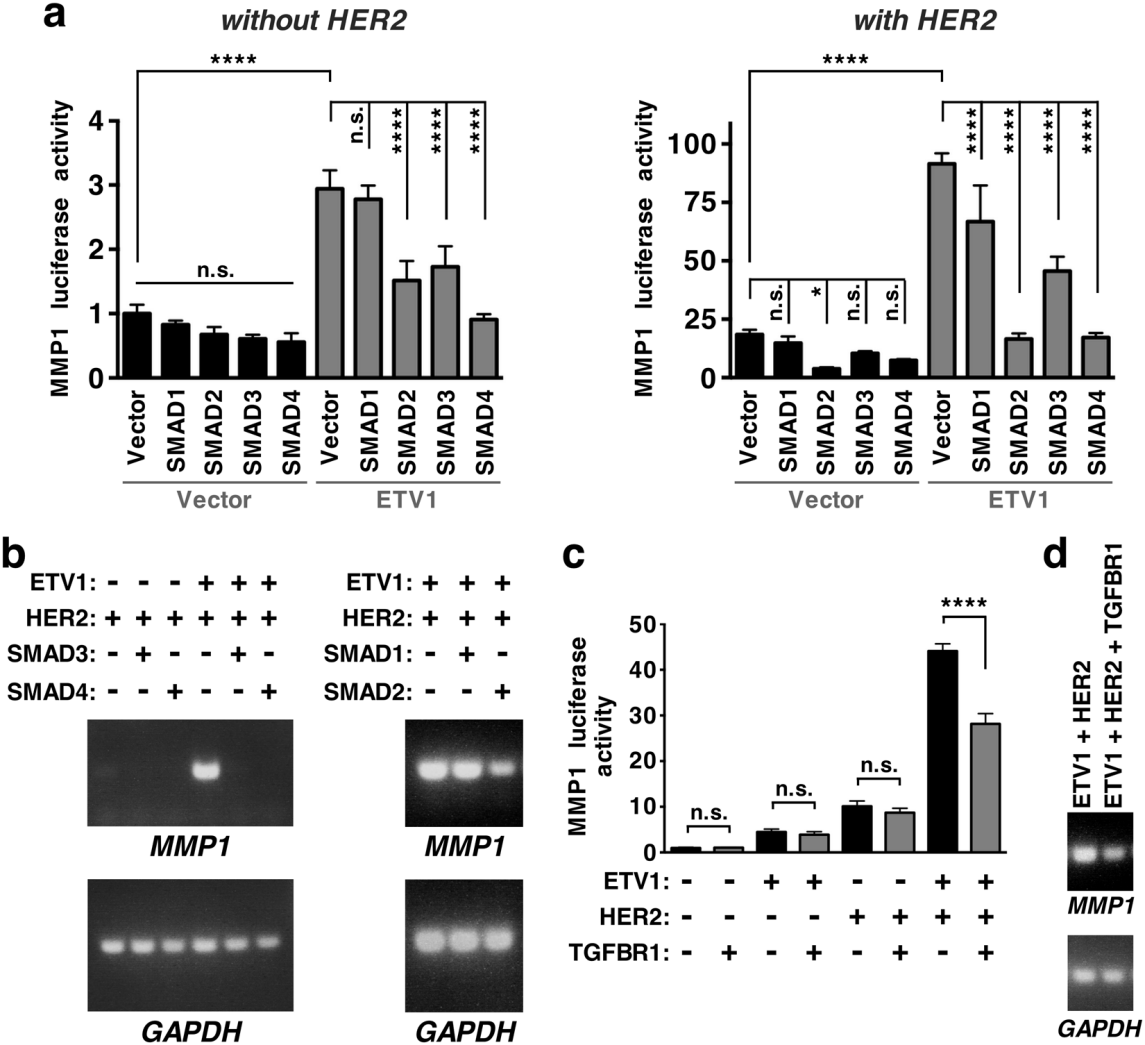
Inhibition of ETV1-mediated transcription by SMAD proteins. (**a**) MMP1 luciferase activity was measured in 293T cells in the presence of indicated proteins. Shown are means (n = 4) with standard deviations. Statistical significance was determined with one-way ANOVA (Tukey’s multiple comparisons test); n.s., not significant; *P < 0.05; ****P < 0.0001. (**b**) RT-PCR measuring endogenous MMP1 gene expression in 293T cells. GAPDH levels were determined to demonstrate that comparable amounts of mRNA were utilized. Full-length agarose gels are presented in Supplementary Fig. S2. (**c**,**d**) Analogous to panels a and b. TGFBR1, constitutively active TGF-β receptor I.

Next, we tested whether transcriptional activity of ETV1 can be modulated by interaction with SMAD proteins. To do so, we first utilized an MMP1 reporter gene in 293T cells and expressed SMAD proteins and/or ETV1 (Fig. 3a, left panel). MMP1 reporter gene activity was increased by overexpression of ETV1 as previously reported^14^, but this was blunted by SMAD4, suggesting that binding to SMAD4 represses the transcriptional activity of ETV1. Interestingly, SMAD2 and SMAD3 also repressed ETV1’s activity although they did not bind ETV1 in our overexpression system as shown in Fig. 1a; please see Discussion for possible explanations. On the other hand, SMAD1, which is not downstream of TGF-β but rather of bone morphogenetic proteins^27^, had no impact on ETV1-dependent transcription (Fig. 3a, left panel). We also assessed MMP1 transcription upon overexpression of oncogenic HER2 that greatly stimulates ETV1 activity^14^. Again, we observed that SMAD2-4 repressed ETV1-dependent activity (Fig. 3a, right panel) and even SMAD1 did so, albeit in the least pronounced manner.

In addition, we tested whether induction of endogenous MMP1 would also be suppressed by SMAD proteins in 293T cells. As previously reported^14^, robust MMP1 mRNA induction by ETV1 was achieved through coexpression of oncogenic HER2. When either SMAD3 or SMAD4 were coexpressed, the ETV1-mediated induction of endogenous MMP1 was suppressed (Fig. 3b, left panels, and Supplementary Fig. S2). Similarly, SMAD2 suppressed ETV1 activity, but SMAD1 did not (Fig. 3b, right panels, and Supplementary Fig. S2). These data largely corroborate our luciferase reporter gene assays and demonstrate that the TGF-β downstream effectors, SMAD2, SMAD3 and SMAD4, can inhibit ETV1-mediated transcription.

To assess the impact of TGF-β signaling on ETV1 activity, we also employed a constitutively activated TGF-β receptor I (TGFBR1). Its coexpression reduced ETV1-dependent, HER2-stimulated MMP1 luciferase reporter gene activity (Fig. 3c) and endogenous MMP1 transcription (Fig. 3d and Supplementary Fig. S2). These data further validate that TGF-β signaling can suppress ETV1’s ability to stimulate gene transcription.

### Impact of TGF-β on ETV1-stimulated invasion by RWPE-1 cells

**Figure 4:**
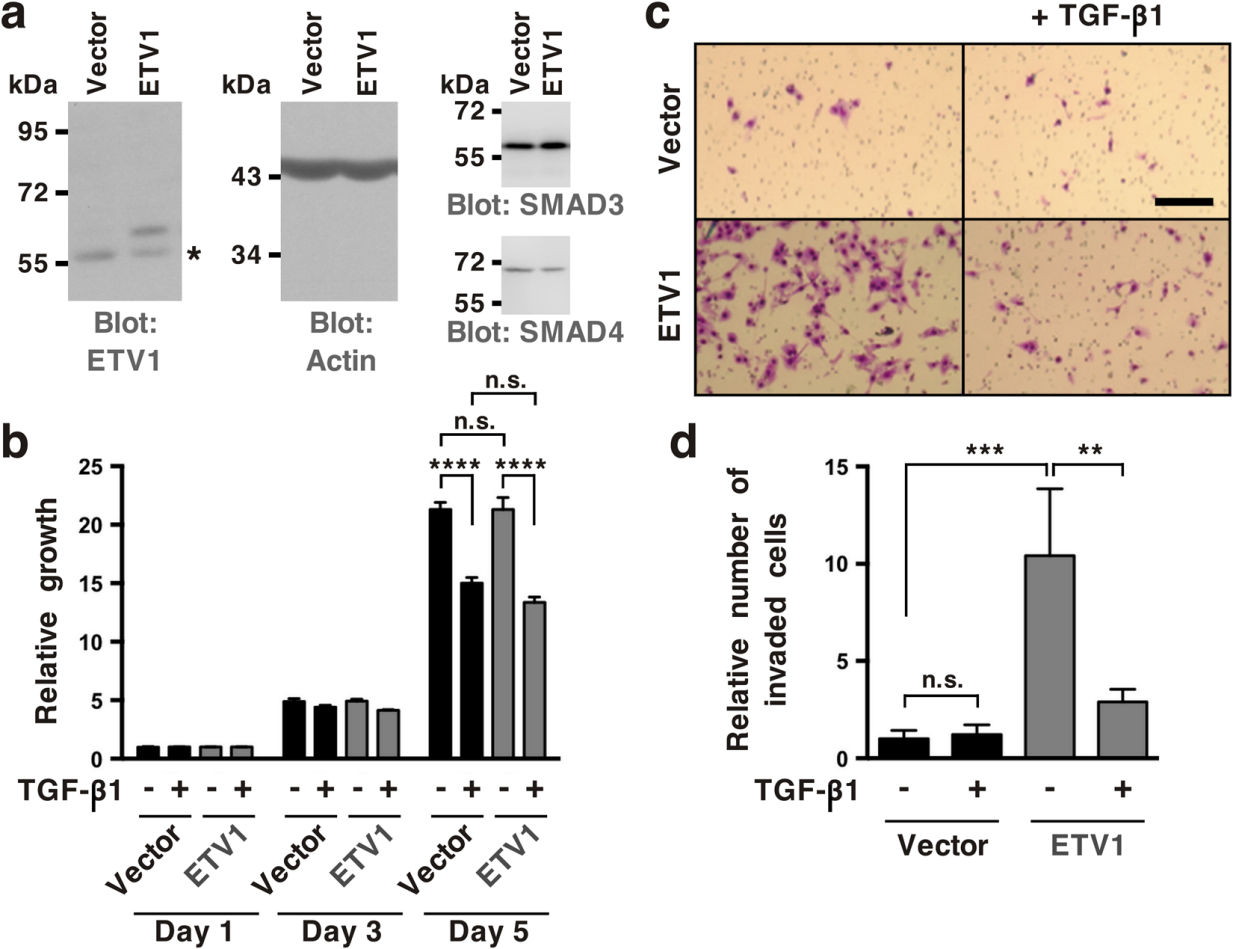
Inhibition of ETV1-dependent invasion by TGF-β1 in RWPE-1 cells. (**a**) Western blot analysis of ETV1 expression in stably transduced RWPE-1 cells. *Non-specific band. ETV1 and actin blots were derived from the same gel that was cut at ~50 kDa and the two resulting parts were then separately processed for western blotting utilizing anti-ETV1 or anti-actin antibodies. SMAD3 and SMAD4 blots were derived from different gels. Full-length blots are presented in Supplementary Fig. S2. (**b**) Cell growth in the presence or absence of TGF-β1 (n = 3). (**c**) Representative images from cell invasion assays. Scale bar = 200 µm. (**d**) Quantification of cell invasion (n = 3). Statistical significance was determined with one-way ANOVA (Tukey’s multiple comparisons test); n.s., not significant; **P < 0.01; ***P < 0.001; ****P < 0.0001. Shown are means with standard deviations.

To assess whether TGF-β signaling modulates physiological effects mediated by ETV1, we utilized benign human prostate RWPE-1 cells and generated respective ETV1 overexpressing stable cells (Fig. 4a and Supplementary Fig. S2); no changes in SMAD3 or SMAD4 protein levels were observable upon ETV1 overexpression. Then, we determined the growth of these cells and found it to be inhibited by TGF-β1, but this was independent of ETV1 overexpression (Fig. 4b). In contrast, cell invasion in the absence of ectopic ETV1 was not affected by TGF-β1, but TGF-β1 suppressed the pro-invasive activity of ETV1 (Fig. 4c,d); this suppression was blunted upon SMAD4 downregulation (Supplementary Fig. S3). These data support the notion that TGF-β signaling can restrain ETV1 activity.

### Impact of ETV1 on TGF-β signaling in PC3 cells

**Figure 5:**
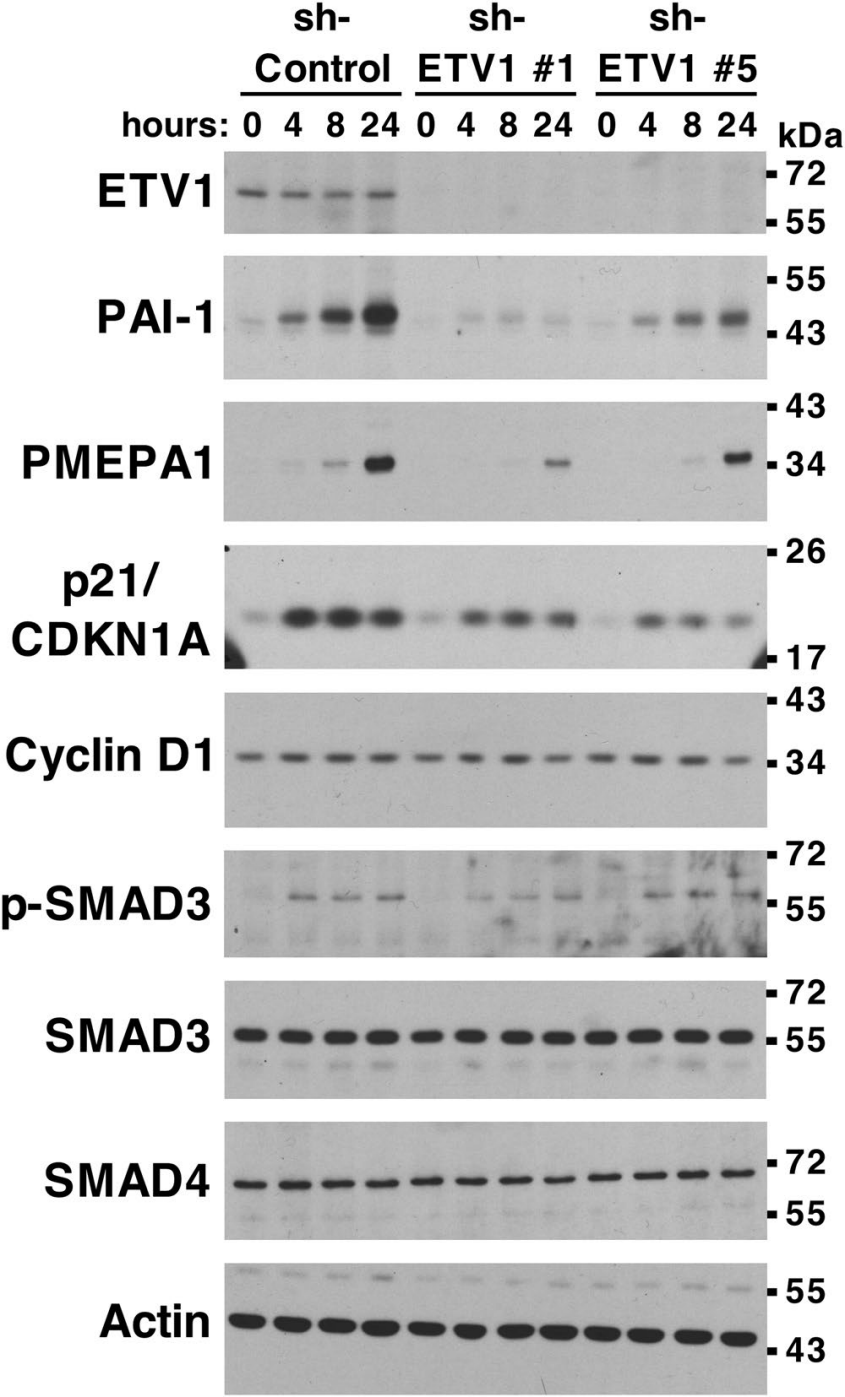
Regulation of TGF-β-dependent gene expression by ETV1. PC3 prostate cancer cells stably expressing control shRNA or two different ETV1 shRNAs were treated with TGF-β1 for 0, 4, 8 or 24 hours. Western blots for indicated proteins are shown. Full-length blots are presented in Supplementary Fig. S4. Blots for PAI-1 and p21 were derived from the same gel cut at ~34 kDa. Similarly, blots for SMAD4 and PMEPA1 were derived from another gel cut at ~43 kDa. A third gel was utilized for cyclin D1 blotting. The ETV1 blot was derived from the stripped PAI-1 blot, the p-SMAD3 blot from the stripped SMAD4 blot, the SMAD3 blot from the stripped cyclin D1 blot, and the actin blot from the stripped SMAD3 blot.

We were curious whether ETV1 might conversely affect TGF-β signaling. To test this, we utilized human PC3 prostate cancer cells, which express high levels of endogenous ETV1 and are not deficient in TGF-β signaling. Thus, we downregulated ETV1 with two different shRNAs, then induced cells with TGF-β1 and measured the expression of known TGF-β-regulated genes, the plasminogen activator inhibitor-1 (PAI-1), the cell cycle inhibitor p21 and the TGF-β signaling regulator PMEPA1^25,26,28^. Of note, ETV1 downregulation suppressed the induction of expression of all these three genes by TGF-β1 (Fig. 5 and Supplementary Fig. S4). No changes of cyclin D1, SMAD3 or SMAD4 protein levels were observed, whereas expectedly phosphorylation of SMAD3 was induced upon TGF-β1 administration and this was not affected by ETV1 shRNAs. These data suggest that, in addition to TGF-β suppressing ETV1 activity, ETV1 can promote TGF-β signaling.

We also overexpressed ETV1 in PC3 cells in order to determine whether this would enhance expression of TGF-β regulated genes. However, this did not affect PAI-1 protein levels upon TGF-β1 administration, yet moderately increased p21 expression (Supplementary Fig. S4), the latter supporting our hypothesis that ETV1 is a promoter of TGF-β signaling. Further, SMAD4 shRNA basically abrogated the induction of PAI-1 and p21 by TGF-β1, confirming that these genes are indeed regulated through SMAD4 (Supplementary Fig. S4). The fact that endogenous ETV1 is already highly expressed in PC3 cells probably accounted for the fact that additional, ectopic ETV1 had no or only a modest effect on TGF-β-mediated gene expression.

### Analysis of *ETV1* transgenic mice and human prostate tumors

**Figure 6:**
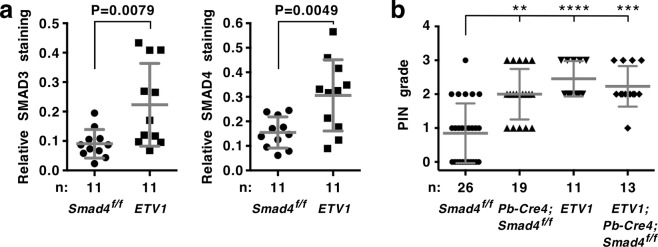
Analysis of *ETV1* transgenic mice at 16.1–25.5 months of age. (**a**) Immunohistochemical analysis of SMAD3 and SMAD4 expression in prostates. *ETV1* transgenic mice were compared to *Smad4*^*f/f*^ mice that are indistinguishable from wild-type mice. Means with standard deviations are shown. Statistical significance was assessed with an unpaired, two-tailed t test. (**b**) Indicated mice were analyzed for PIN formation. Kruskal-Wallis test (Dunn’s multiple comparisons test). **P < 0.01; ***P < 0.001; ****P < 0.0001. Shown are means with standard deviations. Supplementary Fig. S6 provides data for each mouse separately and shows that the average age between the four groups of mice was not statistically different.

To analyze the relationship between ETV1 and SMAD proteins *in vivo*, we harnessed *ETV1* transgenic mice that develop PIN^2,11^. First, we stained their prostates for SMAD3 and SMAD4. While there was little expression of either protein in prostates from control mice (please note that we utilized *Smad4*^*f/f*^ mice as controls), both SMAD3 and SMAD4 levels were enhanced in prostates from age-matched *ETV1* transgenic mice (Fig. 6a and Supplementary Fig. S5). This indicates that SMAD3/4 are present to potentially restrain the oncogenic activity of overexpressed ETV1 in the prostate.

We then asked the question what would happen when SMAD4 becomes ablated. To this end, we crossed transgenic *ETV1* mice with conditional SMAD4 knockout mice (*Smad4*^*f/f*^) and a prostate specific Cre recombinase driver mouse (*Pb-Cre4*). As expected, PIN formation was significantly enhanced in *ETV1* mice (Fig. 6b and Supplementary Fig. S6). Please note that we utilized *Smad4*^*f/f*^ mice as a control and many of them developed low-grade PIN, which is identical to what we observed in old age wild-type mice^17^. Likewise, *Pb-Cre4;Smad4*^*f/f*^ mice displayed significantly enhanced PIN formation, which is in contrast to a previous report^24^ stating that no prostatic lesions were observable. Regardless, *ETV1;Pb-Cre4;Smad4*^*f/f*^ compound mice did not develop prostate adenocarcinomas and the degree of high-grade PIN formation was similar to *ETV1* and *Pb-Cre4;Smad4*^*f/f*^ mice (Fig. 6b and Supplementary Fig. S6). These data suggest that SMAD4 ablation does not promote prostate tumor formation upon ETV1 overexpression (but see Discussion).

**Figure 7:**
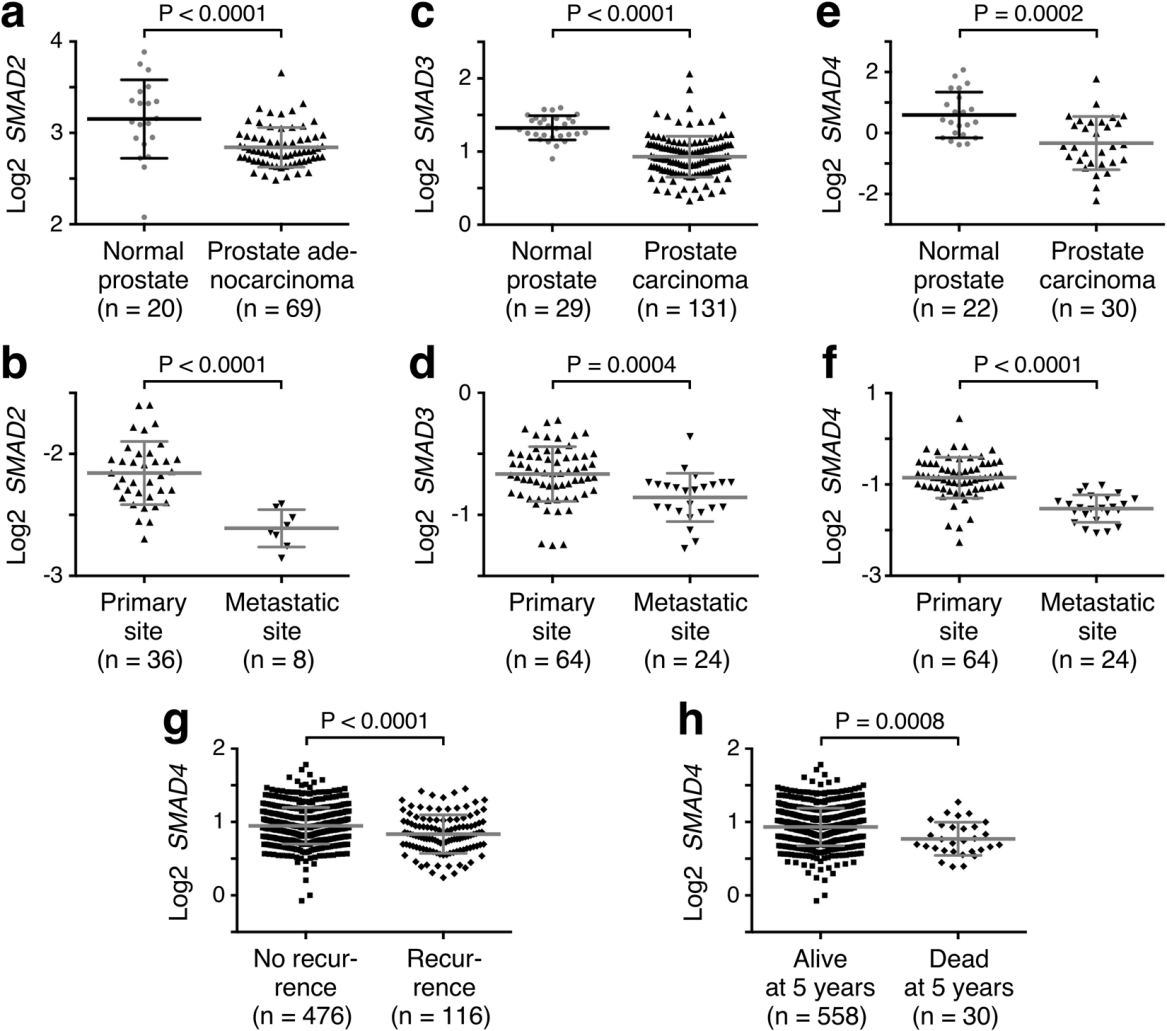
Altered expression of SMAD2, SMAD3 and SMAD4 in prostate cancer. All data were derived with Oncomine from published microarray experiments. (**a**) SMAD2 mRNA levels (probe 203075_at) in normal prostate tissue and prostate adenocarcinoma. Data from Wallace *et al*.^29^. (**b**) SMAD2 mRNA levels (probe 1928_s_at) in prostate tumors at primary and metastatic sites. Data from Holzbeierlein *et al*.^30^. (**c**) SMAD3 mRNA levels (probe 10217) in normal prostate tissue and prostate carcinoma. Data from Taylor *et al*.^31^. (**d**) SMAD3 mRNA levels (probe 38944_at) in prostate tumors at primary and metastatic sites. Data from Yu *et al*.^32^. (**e**) SMAD4 mRNA levels (probe IMAGE:321958) in normal prostate tissue and prostate carcinoma. Data from Tomlins *et al*.^33^. (**f**) SMAD4 mRNA levels (probe 509_at) in prostate tumors at primary and metastatic sites. Data from Yu *et al*.^32^. (**g**) Recurrence and (**h**) survival 5 years after diagnosis are correlated with SMAD4 mRNA levels (probe GI_34147555-S). Data from Nakagawa *et al*.^34^. Shown are means with standard deviations. Number of samples is given in parentheses. Unpaired, two-tailed t test was used to assess statistical significance in all panels.

Lastly, we studied the expression of SMAD2, SMAD3 and SMAD4 in published human microarray data sets^29–34^ with the help of Oncomine (www.oncomine.org). SMAD2 mRNA levels were significantly downregulated in prostate carcinomas compared to normal prostate tissue, and even more so at metastatic compared to primary tumor sites (Fig. 7a,b and Supplementary Fig. S7). The same was true for SMAD3 and SMAD4 (Fig. 7c–f and Supplementary Fig. S7), and low SMAD4 expression was even significantly associated with recurrence and lethality (Fig. 7g,h). Furthermore, SMAD3 and SMAD4 were also downregulated in human prostate tumors being positive for *ETV1* gene fusions (Supplementary Fig. S7). On the other hand, we did not find compelling evidence for SMAD1 downregulation in prostate cancer; rather, SMAD1 mRNA levels may even be upregulated in prostate carcinomas (Supplementary Fig. S7). These bioinformatics results implicate that SMAD2, SMAD3 and SMAD4 may exert tumor suppressive functions in the prostate, including the inhibition of ETV1’s oncogenic activity.

## Discussion

**Figure 8:**
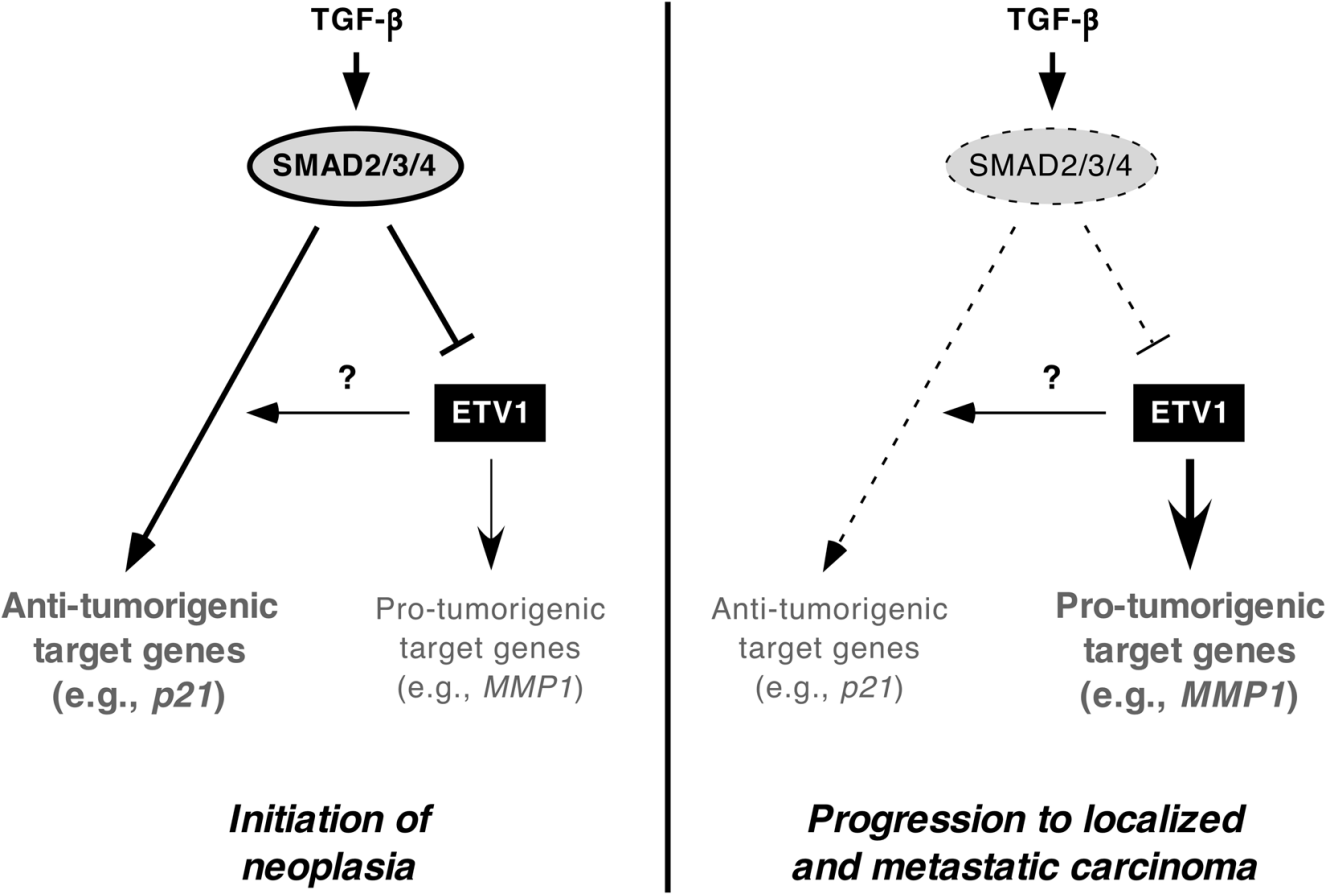
Model showing the relationship between TGF-β/SMAD signaling and ETV1 during prostate tumorigenesis. The mechanism how ETV1 impinges on SMAD-dependent transcription is unknown.

In this report, we identified SMAD4 as a novel interaction partner of ETV1 that can repress ETV1-mediated transcription, providing a mechanism by which TGF-β signaling may constrain ETV1’s oncogenic activity. Furthermore, our data indicate that ETV1 may be needed for maximal TGF-β activity in prostate cancer cells. This suggests a model whereby ETV1 overexpression in the prostate in part limits its own oncogenic potential by activating the tumor suppressive power of TGF-β; but upon SMAD4 (or SMAD2 or SMAD3) downregulation or inactivating mutation, ETV1’s oncogenic activity becomes fully competent and this is needed for the progression of PIN to carcinoma in the prostate (Fig. 8).

Analysis of published microarray data revealed that SMAD2, SMAD3 and SMAD4 mRNA levels decrease during prostate tumorigenesis, implying that loss of their expression could contribute to the progression of PIN to localized carcinoma and then to metastasis. These observations are consistent with previous reports showing that SMAD4 protein and mRNA levels are reduced in prostate carcinomas compared to normal prostate tissue^35–38^. Notably, SMAD3 and likely also SMAD4 interact with the androgen receptor, which may activate or repress androgen-inducible gene transcription in a promoter-dependent manner^39–42^. This interaction with the androgen receptor is mediated through the MH2 domain of SMAD3^39^, whereas the MH2 domain of SMAD4 is dispensable for binding to ETV1. Furthermore, ETV1 is capable of binding to the androgen receptor^11^, which entails ETV1 amino acids 182–477 and thus encompasses amino acids 249–429 that mediate the ETV1-SMAD4 interaction. Hence, it remains to be studied whether SMAD4 and androgen receptor binding to ETV1 are mutually exclusive, or whether a ternary complex can exist.

At present, we do not know how SMAD2 and SMAD3 repressed ETV1-dependent transcription despite the fact that we did not detect coimmunoprecipitation of ETV1 with SMAD2 or SMAD3. However, it is possible that their interaction is of low affinity that precludes detection in coimmunoprecipitation assays. Or alternatively, SMAD4 is needed to facilitate any interaction of ETV1 with SMAD2 and SMAD3, and the amount of endogenous SMAD4 in 293T cells may be too low to result into detectable levels of ETV1-SMAD2/3 complexes. Regardless, the bone morphogenetic protein-regulated SMAD1 transcription factor, which also did not coimmunoprecipitate with ETV1, was incapable of suppressing ETV1-dependent activation of the endogenous MMP1 promoter, implicating that only the TGF-β-regulated SMADs affect ETV1. Interestingly, the bone morphogenetic protein signaling pathway exerts an opposite role to TGF-β signaling in the development of prostate cancer^43^, and maybe the differential functional interaction of ETV1 with SMAD1 and SMAD2/3 is one underlying cause for this.

We attempted to prove that ETV1 overexpression cooperates with SMAD4 loss in the development of prostate cancer by emulating such a situation with our *ETV1;Pb-Cre4;Smad4*^*f/f*^ compound mice. However, these mice were no different from *ETV1* or *Pb-Cre4;Smad4*^*f/f*^ mice and developed only high-grade PIN, but not prostate adenocarcinoma. Similarly, an attempt to show cooperation between ETV1 and JMJD2A, another interaction partner of ETV1, with respective transgenic mice had failed; however, their cooperation in prostate carcinoma formation was revealed in a background of *Pten*^+/−^ mice^17^. Human prostate tumors normally display several mutations at one time^44^, and it has been estimated that three or more driver mutations are required for the development of solid tumors^45^. Accordingly, ETV1 overexpression combined with loss of SMAD4 may be insufficient for carcinoma development, and future studies should focus on the analysis of, for instance, *ETV1;Pb-Cre4;Smad4*^*f/f*^*;Pten*^+/−^ mice to examine whether SMAD4 loss allows overexpressed ETV1 to induce prostate adenocarcinomas in the context of more genetic changes.

Notably, we observed that SMAD3 and SMAD4 protein levels were enhanced in prostates of *ETV1* transgenic mice. This implies that ETV1 overexpression restrains its own oncogenic impact, at least during the initial PIN phase of prostate tumorigenesis, by causing SMAD3/4 overexpression. Although we have not investigated the mechanism, one speculation is that interaction with ETV1 might stabilize SMAD proteins, similar as has been observed for SMAD3 and another ETS transcription factor, the ERG oncoprotein^46^. However, in contradiction to this hypothesis, we observed no alteration of SMAD3/4 protein levels in PC3 prostate cancer cells upon ETV1 downregulation (see Fig. 5) or in RWPE-1 cells upon ETV1 overexpression (see Fig. 4a); this has the caveat that PC3 and RWPE-1 cells are of human and not mouse origin and that *in vitro* cell culture does not always mimic a complex organ such as the prostate. Regardless, this implies the existence of a mechanism different from SMAD stabilization by which ETV1 overexpression could exacerbate, at least in cell culture, TGF-β signaling and its tumor suppressive function. Further, the impact of ETV1 on TGF-β signaling may not be limited to prostate cancer, but pertain to many other normal and diseased tissues where TGF-β exerts important functions during development, homeostasis and pathogenesis.

In conclusion, this study has revealed a novel relationship between ETV1 and TGF-β/SMAD4, which may explain why ETV1 overexpression on its own is insufficient to cause the development of prostate adenocarcinomas. Because ETV1 is not only implicated in prostate cancer, but also many other malignancies such as melanoma, breast and gastrointestinal stromal tumors^47^, the insights provided here will likely have relevance beyond prostate cancer. Lastly, ETV4 and ETV5 are highly homologous to ETV1 and also implicated in prostate cancer development^47,48^. Hence, we predict that ETV4 and ETV5 are also negatively regulated by TGF-β signaling.

## Methods

### DNA constructs, chemicals, enzymes and antibodies

All DNA constructs were made in the corresponding author’s laboratory and verified by DNA sequencing and/or restriction enzyme analysis. Chemicals were purchased at molecular biology or analytical grade purity from established vendors (e.g., Sigma-Aldrich, VWR). Enzymes were obtained from Promega or New England Biolabs. The following antibodies were used for western blotting or immunoprecipitation: anti-Flag M2 (Sigma-Aldrich F1804), anti-Myc 9E10 (Sigma-Aldrich M4439), anti-cyclin D1 DCS6 (Cell Signaling #2926), anti-SMAD4 B-8 (Santa Cruz sc-7966) and anti-actin (GenScript A00730) mouse monoclonal antibodies; anti-ETV1 (Abcam ab81086 or our previously described #959 antibody^9^), anti-p21 H-164 (Santa Cruz sc-756), anti-PMEPA1 2A12 (Abnova H00056937-M01) and anti-SMAD3 (Zymed 51–1500) rabbit polyclonal antibodies; and anti-p-SMAD3-Ser423/425 C25A9 (Cell Signaling #9520) rabbit monoclonal antibodies.

### Coimmunoprecipitation assay

Human embryonic kidney 293T cells were transiently transfected with indicated expression vectors by the calcium phosphate coprecipitation method^49^. Two days later, coimmunoprecipitations were performed essentially as described^50^ employing 50 mM Tris-HCl (pH 7.4), 150 mM NaCl, 50 mM NaF, 0.5% Igepal CA-630, 1 mM PMSF, 10 µg/ml leupeptin, 2 µg/ml aprotinin, 1 µg/ml pepstatin A, 0.1 mM DTT for cell lysis and washing procedures. Coprecipitated proteins were then detected by western blotting^51^.

### Preparation of protein extracts

Human 293T cells were grown in 6-cm plates and transiently transfected by the calcium phosphate coprecipitation method^52^ utilizing 4 µg of 6Myc-tagged ETV1 expression constructs^53^ and 5 µg of pBluescript KS^+^ as a carrier. 36 h after transfection, cells were lysed in 300 µl of 10 mM Tris-HCl, 30 mM Na_4_P_2_O_7_ (pH 7.1), 50 mM NaF, 0.5 mM Na_3_VO_4_, 150 mM NaCl, 1% Triton X-100, 1 mM PMSF, 10 µg/ml leupeptin, 2 µg/ml aprotinin, 1 µg/ml pepstatin A, 1 mM DTT for 30 min on ice. After a clear spin, the supernatants were frozen in liquid nitrogen and stored at −80 °C^54^. Approximately 5–20 µl of these protein extracts were employed for GST pull-down assays.

### GST pull-down assay

Glutathione *S*-transferase (GST) fusion proteins were produced employing standard procedures^55^. Then, GST fusion proteins were bound to 20 µl of glutathione agarose beads in 650 µl of 20 mM HEPES (pH 7.4), 25 mM NaCl, 0.01% Tween-20, 1 mM DTT, 0.4 mM PMSF, 10 µg/ml leupeptin, 2 µg/ml aprotinin, 1 µg/ml pepstatin A for 2 h at 4 °C^56^. Thereafter, beads were washed twice before incubation with protein extracts and 650 µl of the above mentioned binding buffer. 3 h later, beads were washed three times and bound proteins boiled in Laemmli buffer^57^. Finally, SDS polyacrylamide gel electrophoresis was performed and proteins transferred to PVDF membrane^58^. Subsequent incubation with primary and secondary antibodies as well as detection with chemiluminescence was done essentially as described^59^.

### Luciferase assays

Human 293T cells were grown in poly-*L*-lysine coated 12-wells and transiently transfected with 100 ng MMP1-luciferase reporter plasmid^14^, 900 ng pBluescript KS^+^, 5 ng empty vector pEV3S or ETV1 expression plasmid, 10 ng empty vector pEV3S or SMAD expression plasmid, and 2 ng empty vector pQCXIH or pQCXIH-HER2/Neu-V664E expression plasmid utilizing 2 µg polyethylenimine^60^. For Fig. 3c, alternatively 10 ng empty vector pEV3S or ETV1 expression plasmid, 15 ng empty vector pcDNA3 or TGFBR1-T204D (constitutively active TGF-β receptor I) expression plasmid, and 2 ng empty vector pQCXIH or pQCXIH-HER2/Neu-V664E expression plasmid were used. The transfection mixture was washed away with phosphate-buffered saline 8 h later and after another 36 h, cells were lysed and luciferase activities measured as described before^61^.

### RT-PCR

RNA was isolated as described before^62^. This RNA was utilized for cDNA synthesis and amplification by PCR^9^. MMP1 expression was revealed with primers 5′-GTTCAGGGACAGAATGTGCTA-3′ and 5′-CTGCAGTTGAACCAGCTATTAG-3′ that yielded a 350 bp cDNA product. Primers for GAPDH were 5′-GAGCCACATCGCTCAGACACC-3′ and 5′-TGACAAGCTTCCCGTTCTCAGC-3′ (226 bp cDNA product)^63^. Amplified cDNA was revealed after agarose gel electrophoresis by staining with ethidium bromide^64^.

### Cell growth and invasion assays

Retrovirus was produced with the pQCXIN empty vector or pQCXIN-hETV1 expression plasmid as described before^65^. After twice infecting RWPE-1 cells, transduced cells were selected^66^ with 500 µg/ml G418 in keratinocyte serum free media (GIBCO) supplemented with 0.05 mg/ml bovine pituitary extract and 5 ng/ml human recombinant epidermal growth factor. For cell growth assays, 5 × 10^3^ RWPE-1 cells were seeded in 24-well plates and growth was monitored utilizing the PrestoBlue cell viability kit (Invitrogen)^67^ in the presence or absence of TGF-β1 (10 ng/ml). For cell invasion, 5 × 10^4^ RWPE-1 cells (pre-treated with 10 µg/ml mitomycin C for 2 h) were plated on Matrigel invasion chambers (Corning, 8-µm pores) in keratinocyte serum free media containing 0.1% bovine serum albumin in the presence or absence of TGF-β1 (10 ng/ml) and placed into 24-well plates containing keratinocyte serum free media supplemented with 0.05 mg/ml bovine pituitary extract and 5 ng/ml human recombinant epidermal growth factor. After 60 h, non-invaded cells were removed with a cotton swab, invaded cells fixed with methanol, stained with Hemacolor Stain Set (Harleco) and then counted.

### Mouse experiments

All work with mice was approved by the University of Oklahoma Health Sciences Center Institutional Animal Care and Use Committee and was performed in accordance with local and federal guidelines and regulations. Transgenic *ETV1* mice were described before^11^ and maintained on a C57BL/6 background. *Smad4*^*f/f*^ conditional knockout mice (Smad4^tm2.1Cxd^/J; 017462)^68^ were obtained from Jackson Laboratories, while *Pb-Cre4* mice were obtained from the NCI Mouse Repository (B6.Cg-Tg(Pbsn-cre)4Prb/Nci; 01XF5). Please note that only male *Pb-Cre4* mice were utilized for generating the compound mice, since low levels of Cre recombinase expression in oocytes could result in deletion of floxed alleles in all organs. Mice were bred on a mixed background. *ETV1;Pb-Cre4;Smad4*^+*/f*^, *ETV1;Pb-Cre4;Smad4*^*f/f*^ or *Pb-Cre4;Smad4*^*f/f*^ males were bred with *Smad4*^+*/f*^, *Smad4*^*f/f*^ or *ETV1;Smad4*^*f/f*^ females to establish experimental male cohorts. Genotyping of mice was done by standard PCR of tail-snip DNA^69^. To detect the *Pb-Cre4* transgene, primers 5′-CTGAAGAATGGGACAGGCATTG-3′ and 5′-CATCACTCGTTGCATCGACC-3′ (393 bp DNA product) were used. To analyze *Smad4* status, primers 5′-TAAGAGCCACAGGGTCAAGC-3′ and 5′-TTCCAGGAAAAACAGGGCTA-3′ (436 bp for wild-type and ~500 bp for floxed allele) were utilized. Primers for *ETV1* genotyping were described before^11^.

Prostates were fixed with formaldehyde and embedded in paraffin. Then three slides of 4 µm thickness were cut that were 20 µm apart. These slides were stainded with H&E and analyzed for lesions utilizing an established PIN grading system^70^. Scoring was done in a blinded manner. The highest PIN score derived from each of the three corresponding slides was assigned to a specific sample.

### Immunohistochemistry

All staining procedures were performed with a Leica BOND-III machine. An initial 20 min treatment with Bond Epitope Retrieval Solution 1 (Leica Biosystems) was employed, followed by staining with anti-SMAD4 B-8 (Santa Cruz sc-7966) mouse monoclonal antibodies at a 1:200 dilution or with anti-SMAD3 (Zymed 51–1500) rabbit polyclonal antibodies at a 1:250 dilution. After incubation with appropriate secondary antibodies coupled to horseradish peroxidase, 3,3′-diaminobenzidine staining occurred. The stained slides were digitized and digital images extracted with Aperio ImageScope software (Leica). Light transmission was measured with ImageJ software/Fiji (http://fiji.sc) after color deconvolution. Intensity of staining was defined as the difference between the logarithm of maximum light transmission and the logarithm of mean light transmission.

## Supplementary information

Supplementary Information

## Data Availability

Data, detailed protocols and DNA constructs will be made available upon reasonable request. Transgenic *ETV1* mice are not available, because they are no longer maintained; however, the plasmid to generate these transgenic mice is available upon reasonable request.
