# Supplementary material for: Relationship between ETS Transcription Factor ETV1 and TGF-β-regulated SMAD Proteins in Prostate Cancer
Source: Sci Rep. 2019 Jun 3;9:8186. doi: 10.1038/s41598-019-44685-3 (PMC6546734; doi:10.1038/s41598-019-44685-3)
Supplement: Supplementary file 1 — Supplementary Information [file 41598_2019_44685_MOESM1_ESM.pdf]

SUPPLEMENTARY INFORMATION

Relationship between ETS Transcription Factor ETV1 and TGF-β-regulated SMAD Proteins in Prostate Cancer

Sangphil Oh, Sook Shin, Hoogeun Song, Joseph P. Grande & Ralf Janknecht

SUPPLEMENTARY FIGURES

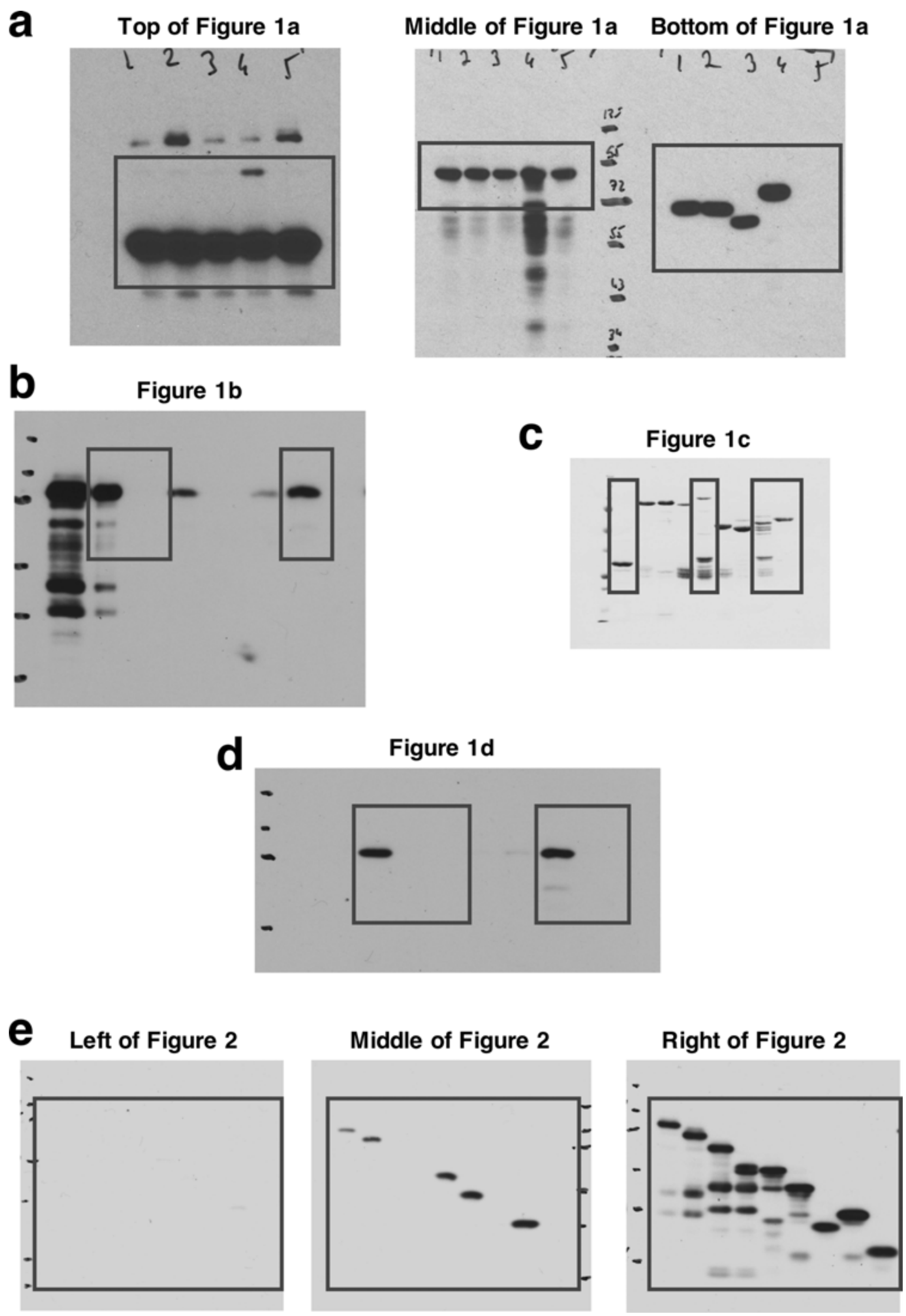

Supplementary Figure S1. Uncropped images for which boxed areas are shown in the main manuscript. (**a**) Corresponding to Figure 1a. (**b**) Corresponding to Figure 1b. (**c**) Corresponding to Figure 1c. (**d**) Corresponding to Figure 1d. (**e**) Corresponding to Figure 2.

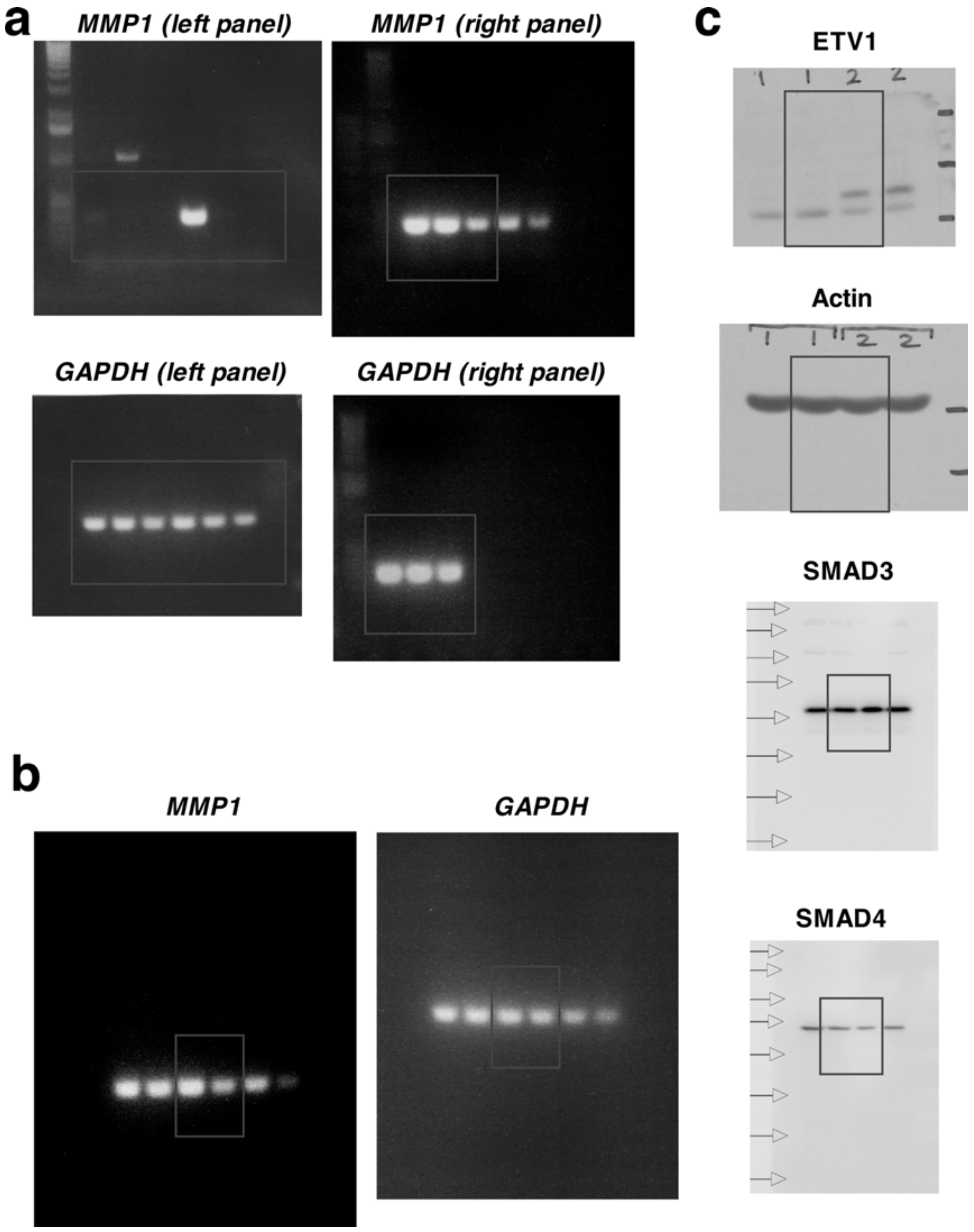

Supplementary Figure S2. Uncropped images for which boxed areas are shown in the main manuscript. (**a**) Corresponding to Figure 3b. (**b**) Corresponding to Figure 3d. (**c**) Corresponding to Figure 4a.

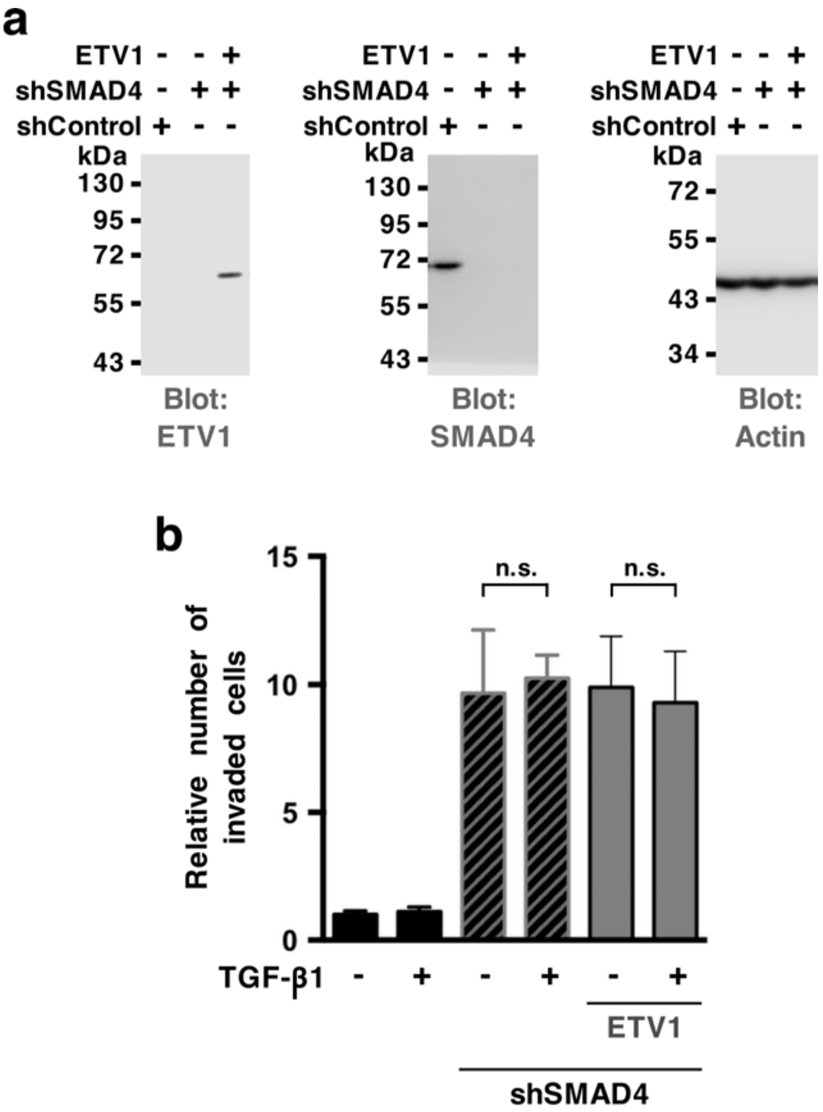

Supplementary Figure S3. Impact of SMAD4 on invasion in RWPE-1 cells. (**a**) Western blots of RWPE-1 cells stably expressing ETV1 and either control shRNA or shRNA directed against SMAD4 (targeting the sequence 5'-GCAGACAGAAACTGGAUUAAA-3'). (**b**) Invasion through Matrigel was measured after 60 hours. Shown are means with standard deviations (n = 4). One-way ANOVA (Tukey's multiple comparisons test) was used to assess statistical significance; n.s., not significant.

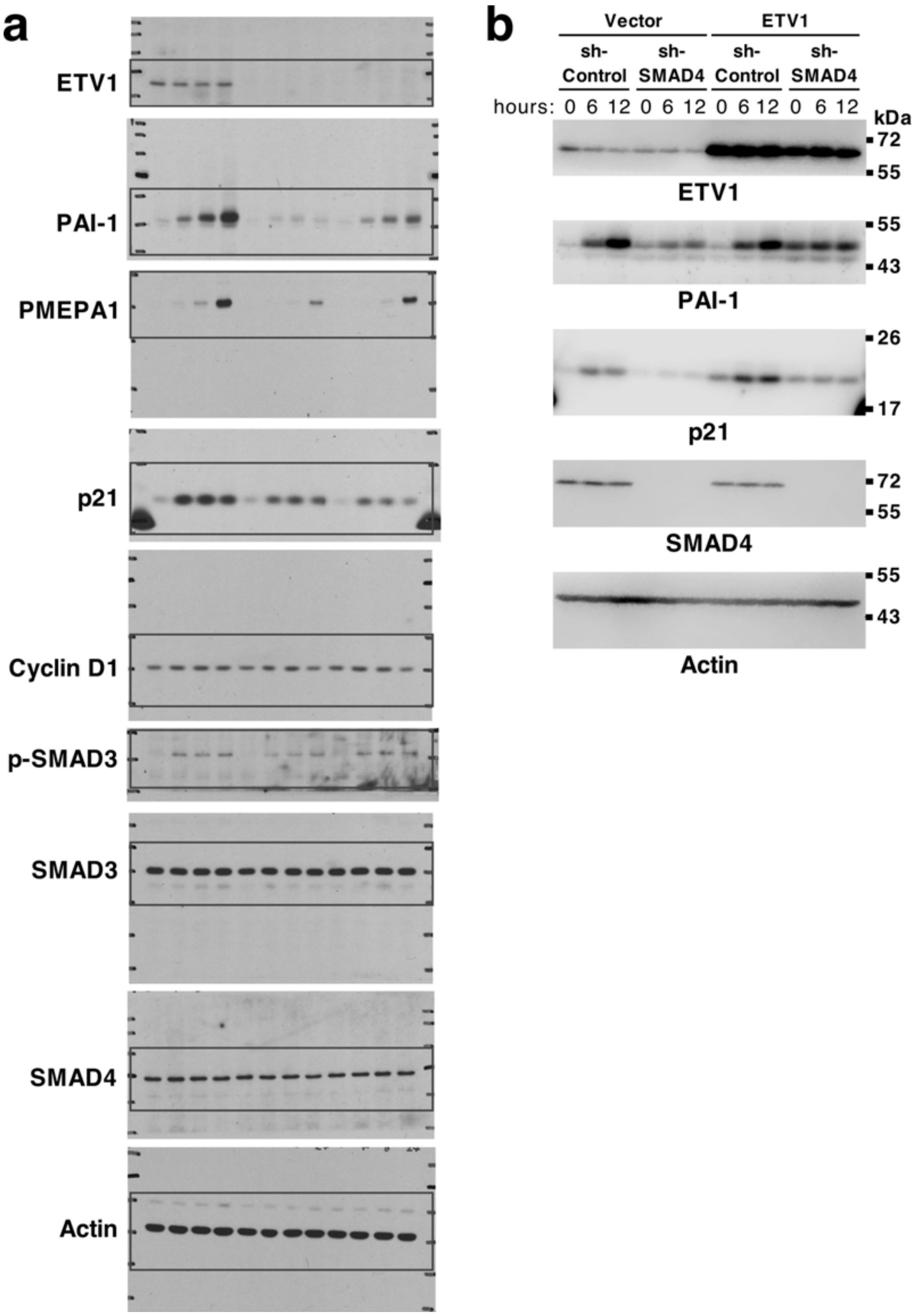

Supplementary Figure S4. (**a**) Uncropped images for which boxed areas are shown in the main manuscript in Figure 5. (**b**) PC3 prostate cancer cells expressing control or SMAD4 shRNA and furthermore ectopic ETV1 were treated with TGF-β1 for 0, 6 or 12 hours. Western bots for ETV1 and PAI-1 were derived from the same gel cut at ~55 kDa. Blots for p21 and SMAD4 were derived from other gels. The actin blot was derived from the stripped PAI-1 blot.

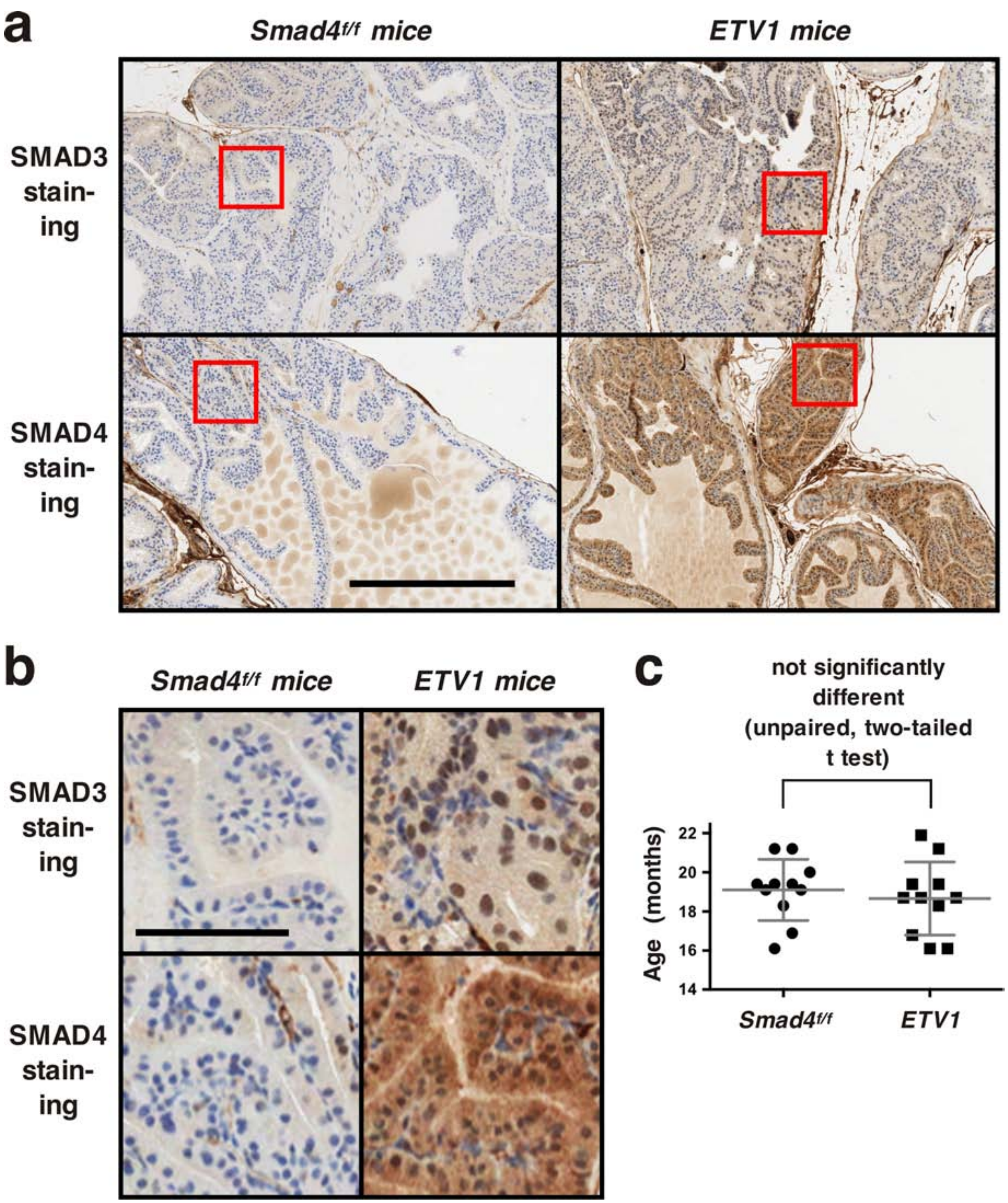

Supplementary Figure S5. (**a**) Representative images showing immunohistochemical staining of *ETV1* and *Smad4*$^{f/f}$ prostates. Scale bar = 500 µm. (**b**) Larger magnification of red-marked areas in panel "a". Scale bar = 100 µm. (**c**) Age distribution of mice utilized in Figure 6a.

a

ID	Genotype	Age (months)	PIN Grade
77	***Smad4*$^{f/f}$**	16.1	0
507	***Smad4*$^{f/f}$**	16.9	0
509	*Smad4*$^{f/f}$	18.3	0
511	***Smad4*$^{f/f}$**	18.3	0
882	*Smad4*$^{f/f}$	18.7	1
708	***Smad4*$^{f/f}$**	19.1	0
485	***Smad4*$^{f/f}$**	19.1	1
486	*Smad4*$^{f/f}$	19.1	2
500	***Smad4*$^{f/f}$**	19.4	0
346	*Smad4*$^{f/f}$	19.4	1
340	***Smad4*$^{f/f}$**	19.4	1
341	***Smad4*$^{f/f}$**	19.4	2
240	***Smad4*$^{f/f}$**	20.0	2
131	*Smad4*$^{f/f}$	20.1	2
245	*Smad4*$^{f/f}$	20.1	1
248	*Smad4*$^{f/f}$	20.1	1
258	***Smad4*$^{f/f}$**	21.2	0
260	***Smad4*$^{f/f}$**	21.2	1
789	*Smad4*$^{f/f}$	22.0	0
790	*Smad4*$^{f/f}$	22.0	0
197	*Smad4*$^{f/f}$	22.2	0
382	*Smad4*$^{f/f}$	23.8	0
553	*Smad4*$^{f/f}$	24.7	2
774	*Smad4*$^{f/f}$	24.9	1
783	*Smad4*$^{f/f}$	25.0	1
784	*Smad4*$^{f/f}$	25.0	3
820	*Pb-Cre4;Smad4*$^{f/f}$	16.1	1
78	*Pb-Cre4;Smad4*$^{f/f}$	16.1	2
713	*Pb-Cre4;Smad4*$^{f/f}$	19.1	2
706	*Pb-Cre4;Smad4*$^{f/f}$	19.2	1
506	*Pb-Cre4;Smad4*$^{f/f}$	19.3	2
501	*Pb-Cre4;Smad4*$^{f/f}$	19.4	2
338	*Pb-Cre4;Smad4*$^{f/f}$	19.4	3
682	*Pb-Cre4;Smad4*$^{f/f}$	19.5	3
683	*Pb-Cre4;Smad4*$^{f/f}$	19.5	2
132	*Pb-Cre4;Smad4*$^{f/f}$	20.1	1
134	*Pb-Cre4;Smad4*$^{f/f}$	20.1	1
246	*Pb-Cre4;Smad4*$^{f/f}$	20.1	2
161	*Pb-Cre4;Smad4*$^{f/f}$	21.4	3
162	*Pb-Cre4;Smad4*$^{f/f}$	21.4	2
193	*Pb-Cre4;Smad4*$^{f/f}$	22.2	2
544	*Pb-Cre4;Smad4*$^{f/f}$	24.8	1
781	*Pb-Cre4;Smad4*$^{f/f}$	25.0	3
753	*Pb-Cre4;Smad4*$^{f/f}$	25.5	3
754	*Pb-Cre4;Smad4*$^{f/f}$	25.5	2
818	****ETV1***	16.1	2
281	****ETV1***	16.1	3
277	****ETV1***	16.8	3
514	****ETV1***	18.3	3
268	****ETV1***	18.7	3
274	****ETV1***	18.7	2
275	****ETV1***	18.7	3
503	****ETV1***	19.4	2
337	****ETV1***	19.4	2
261	****ETV1***	21.2	2
97	****ETV1***	21.9	2
817	*ETV1;Pb-Cre4;Smad4*$^{f/f}$	16.1	2
819	*ETV1;Pb-Cre4;Smad4*$^{f/f}$	16.1	2
512	*ETV1;Pb-Cre4;Smad4*$^{f/f}$	18.3	1
267	*ETV1;Pb-Cre4;Smad4*$^{f/f}$	18.7	2
712	*ETV1;Pb-Cre4;Smad4*$^{f/f}$	19.1	2
487	*ETV1;Pb-Cre4;Smad4*$^{f/f}$	19.1	3
499	*ETV1;Pb-Cre4;Smad4*$^{f/f}$	19.4	2
504	*ETV1;Pb-Cre4;Smad4*$^{f/f}$	19.4	2
339	*ETV1;Pb-Cre4;Smad4*$^{f/f}$	19.4	2
687	*ETV1;Pb-Cre4;Smad4*$^{f/f}$	19.5	3
259	*ETV1;Pb-Cre4;Smad4*$^{f/f}$	21.2	2
98	*ETV1;Pb-Cre4;Smad4*$^{f/f}$	21.9	3
755	*ETV1;Pb-Cre4;Smad4*$^{f/f}$	25.5	3

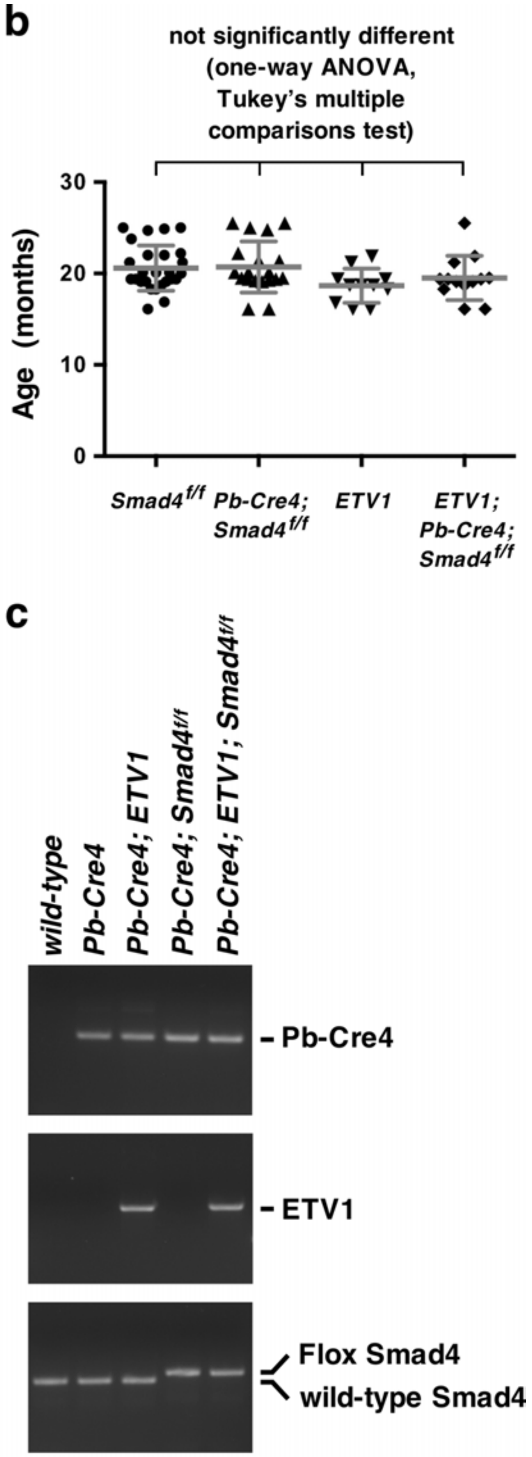

Supplementary Figure S6. (**a**) Age and PIN grade of mice. Asterisks mark mice utilized in Figure 6a for immunostaining. (**b**) Age distribution of mice utilized in Figure 6b. (**c**) Representative genotyping of recombinant mice. Shown are PCR products indicating the presence or absence of the *Pb-Cre4* or *ETV1* transgene or the status at the *Smad4* locus (wild-type or floxed allele). Please note that genotyping was done on DNA from tails and thus does not reveal any Cre-mediated knockout of *Smad4* in the prostate.

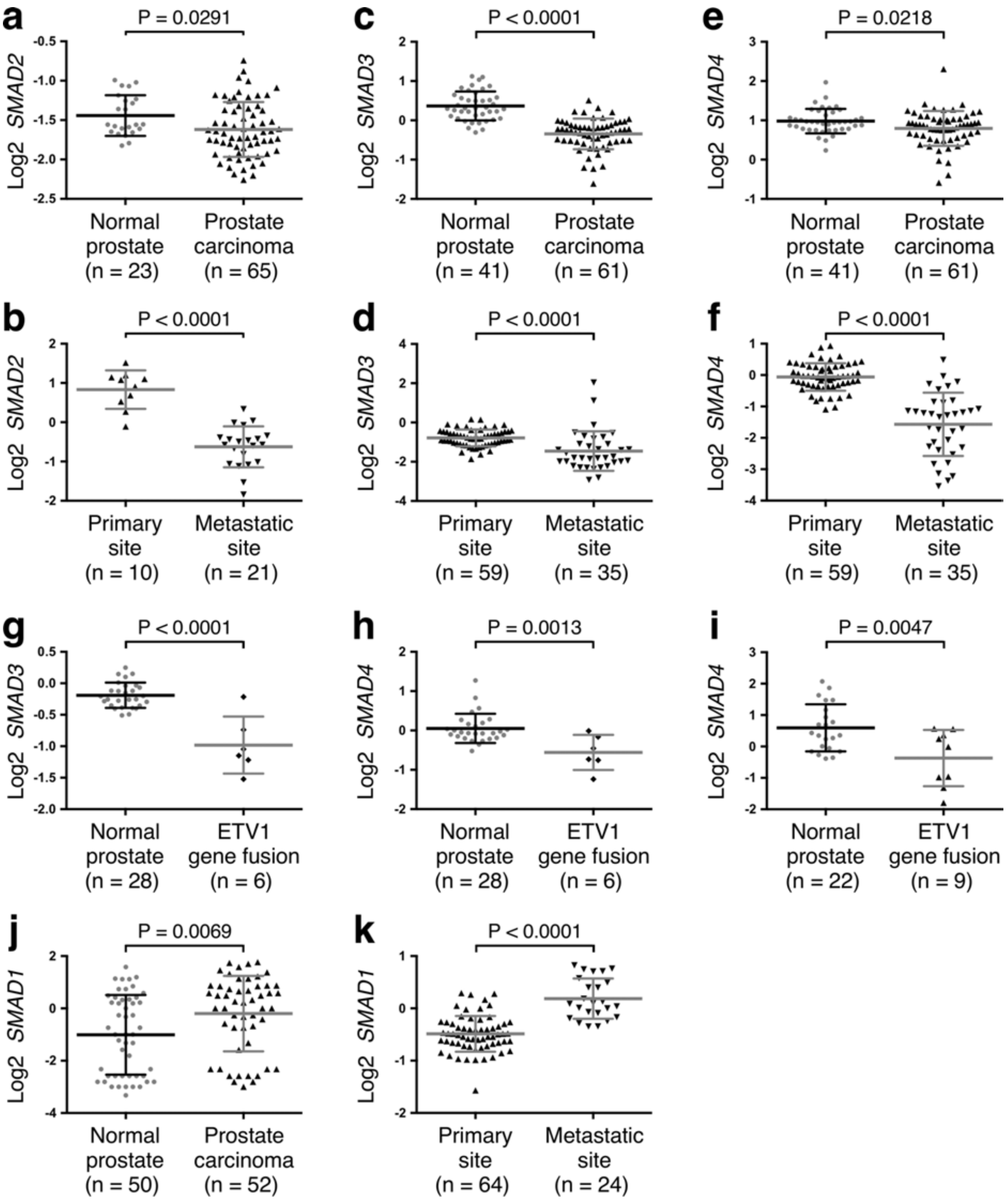

Supplementary Figure S7. SMAD1-4 mRNA levels in prostate cancer. (**a**) SMAD2 mRNA levels (probe 1928_s_at) in normal prostate tissue and prostate carcinoma. Data from Yu *et al* [1]. (**b**) SMAD2 mRNA levels (probe GE59354) in prostate tumors at primary and metastatic sites. Data from Chandran *et al* [2]. (**c**) SMAD3 mRNA levels (probe IMAGE:323028) in normal prostate tissue and prostate carcinoma. Data from Lapointe *et al* [3]. (**d**) SMAD3 mRNA levels

(probe A_23_P359091) in prostate tumors at primary and metastatic sites. Data from Grasso *et al* [4]. (**e**) SMAD4 mRNA levels (probe IMAGE:788421) in normal prostate tissue and prostate carcinoma. Data from Lapointe *et al* [3]. (**f**) SMAD4 mRNA levels (probe A_23_P27346) in prostate tumors at primary and metastatic sites. Data from Grasso *et al* [4]. (**g**) SMAD3 mRNA levels (probe A_23_P359091) in normal prostate tissues and ETV1 fusion gene-positive prostate carcinomas (primary and metastatic sites). Data from Grasso *et al* [4]. (**h**) SMAD4 mRNA levels (probe A_23_P27346) in normal prostate tissues and ETV1 fusion gene-positive prostate carcinomas (primary and metastatic sites). Data from Grasso *et al* [4]. (**i**) SMAD4 mRNA levels (probe IMAGE:321958) in normal prostate tissues and ETV1 fusion gene-positive prostate carcinomas (primary and metastatic sites). Data from Tomlins *et al* [5]. (**j**) SMAD1 mRNA levels (probe 37280_at) in normal prostate tissue and prostate carcinoma. Data from Singh *et al* [6]. (**k**) SMAD1 mRNA levels (probe 37280_at) in prostate tumors at primary and metastatic sites. Data from Yu *et al* [1]. Shown are means with standard deviations. Number of samples is given in parentheses. Unpaired, two-tailed t test was used.

SUPPLEMENTARY REFERENCES

1. Yu YP, Landsittel D, Jing L, Nelson J, Ren B, Liu L, McDonald C, Thomas R, Dhir R, Finkelstein S, Michalopoulos G, Becich M, Luo JH (2004) Gene expression alterations in prostate cancer predicting tumor aggression and preceding development of malignancy. *J. Clin. Oncol.* **22**, 2790-2799.
2. Chandran UR, Ma C, Dhir R, Bisceglia M, Lyons-Weiler M, Liang W, Michalopoulos G, Becich M, Monzon FA (2007) Gene expression profiles of prostate cancer reveal involvement of multiple molecular pathways in the metastatic process. *BMC Cancer* **7**, 64.
3. Lapointe J, Li C, Higgins JP, van de Rijn M, Bair E, Montgomery K, Ferrari M, Egevad L, Rayford W, Bergerheim U, Ekman P, DeMarzo AM, Tibshirani R, Botstein D, Brown PO, Brooks JD, Pollack JR (2004) Gene expression profiling identifies clinically relevant subtypes of prostate cancer. *Proc. Natl. Acad. Sci. USA* **101**, 811-816.
4. Grasso CS, Wu YM, Robinson DR, Cao X, Dhanasekaran SM, Khan AP, Quist MJ, Jing X, Lonigro RJ, Brenner JC, Asangani IA, Ateeq B, Chun SY, Siddiqui J, Sam L, Anstett M, Mehra R, Prensner JR, Palanisamy N, Ryslik GA, Vandin F, Raphael BJ, Kunju LP, Rhodes DR, Pienta KJ, Chinnaiyan AM, Tomlins SA (2012) The mutational landscape of lethal castration-resistant prostate cancer. *Nature* **487**, 239-243.
5. Tomlins SA, Mehra R, Rhodes DR, Cao X, Wang L, Dhanasekaran SM, Kalyana-Sundaram S, Wei JT, Rubin MA, Pienta KJ, Shah RB, Chinnaiyan AM (2007) Integrative molecular concept modeling of prostate cancer progression. *Nat. Genet.* **39**, 41-51.
6. Singh D, Febbo PG, Ross K, Jackson DG, Manola J, Ladd C, Tamayo P, Renshaw AA, D'Amico AV, Richie JP, Lander ES, Loda M, Kantoff PW, Golub TR, Sellers WR (2002) Gene expression correlates of clinical prostate cancer behavior. *Cancer Cell* **1**, 203-209.
